# Studies on the Microstructural Evolution and Mechanical Properties of Superalloy Inconel 718 Induced by Low Plasticity Burnishing Coupled with Turning

**DOI:** 10.3390/ma15113740

**Published:** 2022-05-24

**Authors:** Yang Hua, Zhanqiang Liu, Jie Yi, Aijun Tang

**Affiliations:** 1 School of Mechanical and Electronic Engineering, Shandong Jianzhu University, Jinan 250101, China; tajsmile@sdjzu.edu.cn; 2 Key Laboratory of High Efficiency and Clean Mechanical Manufacture, Ministry of Education, School of Mechanical Engineering, Shandong University, Jinan 250061, China; melius@sdu.edu.cn; 3 Key National Demonstration Center for Experimental Mechanical Engineering Education, School of Mechanical Engineering, Shandong University, Jinan 250061, China

**Keywords:** low plasticity burnishing, microstructure, residual stress, mechanical properties, Inconel 718

## Abstract

Mechanical surface treatments are needed to perform on components for fatigue life enhancement by introducing beneficial compressive residual stress and material strengthening. In this study, the combined turning with low plasticity burnishing (LPB) surface modification process was performed for the sake of improving mechanical properties of Inconel 718. Firstly, the evolution of microstructure and residual stress after the LPB process were analyzed with the aid of electron backscatter diffraction (EBSD) and X-ray diffraction (XRD), respectively. Secondly, the tensile behavior of treated samples was investigated through tension tests. Finally, the micro-strengthening mechanism of Inconel 718, induced by the LPB process, was revealed. The results show that the peak compressive stress is increased by a factor of 4.2 after the LPB process. The grain refinement induced by the LPB process is attributed to the increase of average misorientation and the formation of high angle grain boundaries (HAGBs). The enhanced yield strength depends on the decreased average spacing and the increased HAGBs.

## 1. Introduction

Inconel 718 has excellent properties including high strength, corrosion resistance and good fatigue behavior at an elevated temperature of 650 °C. Hence, it is extensively used in aerospace industries for turbine disks, shafts, and blades [1,2,3]. However, Inconel 718 is a typical difficult-to-cut material due to poor machinability, which accompanies high temperature [4], the evolution of microstructure, the formation of large tensile residual stress, cracks and other defects. Such phenomena might provoke several problems, such as the degradation of mechanical property and the premature fatigue failure of components [5].

In order to improve the fatigue performance of components, surface integrity must be enhanced by introducing compressive residual stress, raising the mechanical property and decreasing the roughness. Some attempts have been conducted, including the application of coatings [6] and the formation of diffusion layers [7]. Although these methods have the ability to improve the surface properties, the issues related to adhesion of the improved layer to the metallic substrate are the main limitation.

On the other hand, mechanical surface modification technologies are commonly performed to introduce beneficial compressive residual stresses and surface strengthening for fatigue life improvement, including shot peening, low plasticity burnishing and laser shock peening [8,9]. Shot peening (SP) typically produces a high degree of cold-work (up to 30–40%) and compressive residual stress. However, the surface roughness generated by shot peening is much higher than the requirement of turbine components. In addition, the large degree of cold-work can speed up the relaxation rate of compressive residual stress [10]. Laser shock peening (LSP) is another common surface treatment process for nickel-based superalloys. In comparison to SP process, LSP process induces a deeper depth of compressive residual stress (up to 2 mm) and a lesser degree of cold-work (7–15%) [11,12]. Nevertheless, the surface finish of Inconel 718 produced by LSP is poor, and the LSP is relatively costly due to the expensive equipment and coatings [8,11].

Low plasticity burnishing (LPB) is a cost-effective surface treatment process which employs spherical ball rolling on the material surface to produce plastic deformation. The LPB process can provide a smoother surface, deeper compressive residual stress layer and a lesser degree of cold-work compared with SP and LSP, while inhibiting the crack initiation and propagation [13,14]. Therefore, the advantages of LPB have attracted the interest of researchers and engineers. Raaj et al. [15] investigated the surface mechanical behavior of low plasticity burnished Inconel 718. They highlighted that decreased porosity, and increased compressive residual stresses and microhardness were observed when the burnishing pressures were increased from 10 MPa to 40 MPa. The burnishing induced mechanical loads of a higher order resulted in severe plastic deformation in the subsurface region, which reduced microporosity and increased compressive residual stresses. Miranda et al. [16] compared the effects of three different mechanical finishing processes on the surface properties of Inconel 718, including polishing, ball burnishing, and hammer-peening. The results revealed that the burnishing process produced the lowest roughness (Ra = 0.17 μm) and the thickest compressive layer in comparison to polishing and hammer-peening processes. Okada et al. [17] investigated the effect of ball burnishing on surface roughness and hardness of a nickel-based superalloy. They found that the surface hardness increased with the increase of burnishing pressure, the subsurface microstructure was also changed in the region of approximately 20 μm, and the lower surface roughness was produced as the pressure increased from 20 MPa to 60 MPa. The decreased surface roughness could be attributed to the disappearance of grooves when the burnishing pressure increased.

Some researchers studied the evolution of microstructure after the burnishing process. Pu et al. [18] found that the grain size reduced significantly from 11.4 μm to 1.4 μm, and strong basal textures have been created after burnishing AZ31B magnesium alloy; a similar phenomenon was also observed by Grzesik et al. [19] for 41Cr4 steel. The mechanism of dynamic recrystallization has been introduced by Pu et al. [18] to explain this subsurface microstructure. Delgado et al. [20] described the phenomenon of the grain refinement, the micro-twins, the increment of dislocation density and the dislocation of cell structures near the burnished surface of different materials. However, most of these investigations focused on aluminum alloys [21], titanium alloys [22,23] or steels [24]. The evolution of microstructure after low plasticity burnished Inconel 718 has rarely been studied previously, despite Kumar et al. [25] reporting that microstructure was crucial to improve fatigue behavior.

In accordance with the aforementioned, it can be found that the current studies have been concentrating on surface integrity, such as surface roughness and microhardness induced by the LPB process. However, the microstructural evolution of Inconel 718 after the LPB process has rarely been reported at present. In addition, the micro-strengthening mechanism of Inconel 718 induced by the LPB process has not yet been revealed. Therefore, the present work aims to fill this gap by investigating the evolution of microstructure and mechanical properties. In the present sturdy, the microstructural characteristics such as misorientation distribution, grain size and interface boundaries are especially emphasized. The distribution of residual stress and plastic strain are analyzed after the LPB process. Moreover, the strengthening mechanism of Inconel 718, induced by the LPB process, is also detailed analyzed and revealed. The results of this study will be beneficial to guide the actual machining process of turbine disks and turbine shafts in aero-engine factories.

## 2. Experiments

### 2.1. Material

**Figure 1:**
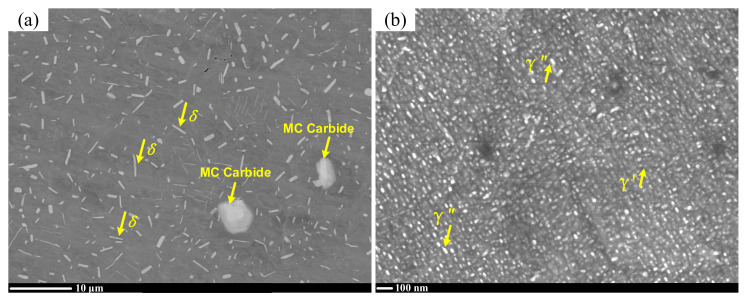
Microstructure of Inconel 718 at (**a**) medium and (**b**) high magnification scanning electron microscopy (SEM).

The nickel-based superalloy Inconel 718 was employed in the present study. The as-received material had undergone forging, solution treatment (heat treated at 960 °C for 1 h, air-cooled to room temperature) and aging treatment (heat treated at 720 °C for 8 h, then furnace cooled to 620 °C and kept it for 8 h, finally air-cooled to room temperature). The chemical compositions of Inconel 718 were measured by electron dispersive spectroscopy (EDS) (Oxford, UK), as shown in Table 1. The microstructure of Inconel 718 was observed by scanning electron microscope (SEM) (JEOL, Akishima, Japan), as presented in Figure 1. Large primary MC type carbides can be observed (see Figure 1a), which were formed during the solution treatment.

### 2.2. LPB Process and Tensile Test

**Figure 2:**
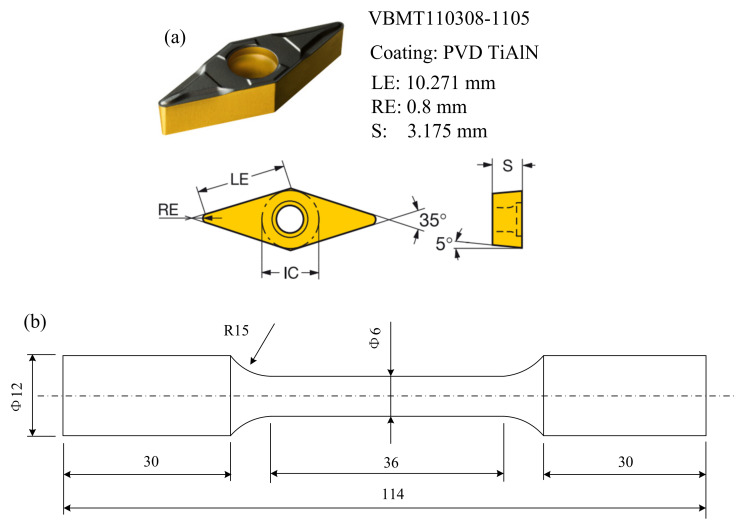
FT experiments: (**a**) the geometry of carbide insert; (**b**) the shape and dimensions of specimen (all dimensions in millimetres).

In order to understand the microstructure, plastic strain and mechanical properties evolution, two integrated processes were employed: finish turning (FT), and FT followed by LPB. Finish turning experiments were carried out on a CNC turning center (Daewoo, Seoul, Korea). The carbide inserts ISO VBMT110308-1105 (Sandvik, Stockholm, Sweden) with PVD coating (TiAlN) and a tool holder ISO SVJBR 2525M 11 (Sandvik, Stockholm, Sweden) were employed for turning Inconel 718. The turning parameters (cutting speed, feed rate, the depth of cut) were determined, taking into account the tool maker recommendations for typical and industrial machining operations for nickel-based alloys. Thus, the FT experiments were conducted at the following parameters: cutting speed *V* = 60 m/min, feed rate *f* = 0.1 mm/rev, depth of cut *a_p_* = 0.2 mm, tool nose radius RE = 0.8 mm. The geometry of the carbide insert was shown in Figure 2a. The shape and dimensions of the specimen after the FT process was shown in Figure 2b.

**Figure 3:**
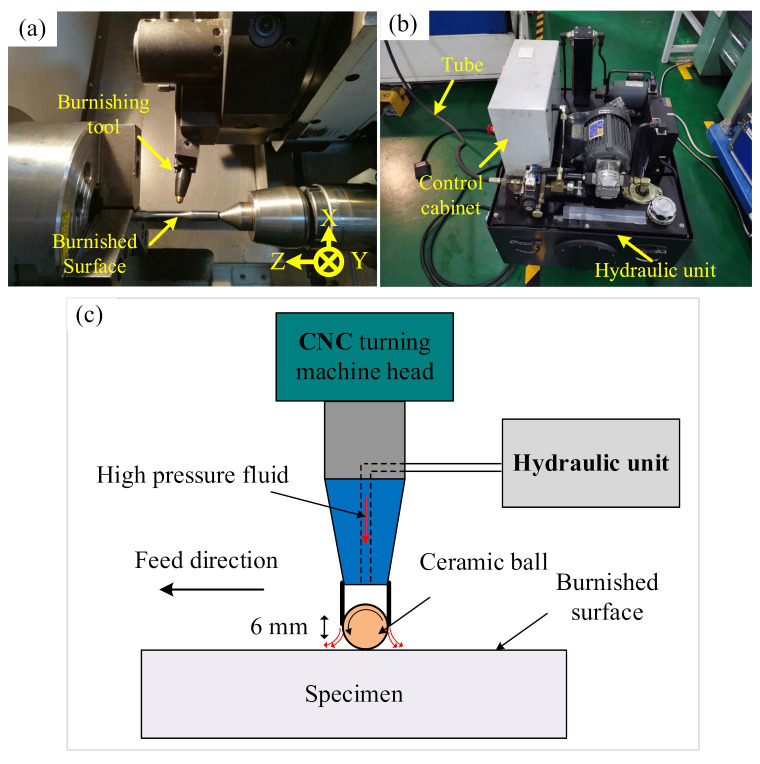
LPB experimental set-up: (**a**) LPB process on the CNC turning center; (**b**) hydraulic unit; (**c**) principle of LPB process.

LPB experiments were applied on the FT specimens by using a ceramic ball with a diameter of 6 mm, as illustrated in Figure 3. A ceramic ball made of silicon nitride was quipped on the tip of the burnishing tool. This ceramic ball was hydrostatically loaded on the workpiece surface by a hydraulic unit which produced the pressure at the range of 0~20 MPa, which plastically deforms the peak of roughness and moves the material to the ‘‘valleys’’ of the roughness profile. The LPB experiments were performed on the same CNC turning center (Daewoo, Seoul, Korea) at the following parameters: burnishing pressure *P* = 18 MPa, the number of passes *n* = 2, burnishing speed *V_LPB_* = 60 m/min, burnishing feed rate *f_LPB_* = 0.1 mm/rev. All the specimens were fabricated at the same process parameters to ensure the similar plastic deformation and mechanical properties. A tension testing machine (Zwic-Z250) was employed to perform the static tension tests at room temperature according to ASTME8/E8M (Standard Test Methods for Tension Testing of Metallic Materials) [26]. The tension tests were conducted with cross-head speed control at a rate of 0.015 mm/min for the sake of determining the yield and ultimate strength properly.

### 2.3. Measurements

Electron back-scatter diffraction (EBSD) technique was employed to analyze the change of grain size and misorientation in the surface layer. For EBSD observation, transverse sections perpendicular to the finish turned and low plasticity burnished surface were made from the samples. These transverse sections were ground and polished first, then Ar-ion milling was used to make the transverse section smoother than the polished surface. The JEOL JSM-7800F (JEOL, Akishima, Japan) system equipped with Channel 5 software was utilized to collect and analyz EBSD patterns. A scanning area of 200 × 200 μm from the surface with a step of size of 0.5 μm was operated at 20 kV for finish turned and low plasticity burnished specimens.

Residual stresses were measured by using X-ray diffraction (XRD) with the cosα method [27]. A *μ*-X360n XRD system (Pulstec, Hamamatsu, Japan) with Cr-Kβ radiation line was employed to measure residual stress at the crystal plane {311} of Inconel 718. The parameters for measurements were summarized in Table 2. In order to obtain the residual stress in depth, electro-polishing (electrolyte: 12.5 vol% sulfuric acid and 87.5 vol% methanol) was used to remove the material layer by layer. Residual stresses in longitudinal and transverse directions were measured at each depth. The full width at half maximum (FWHM) was measured by using the XRD technique to evaluate the plastic strain after FT and LPB treatments.

Hardness was measured at the cross-section of specimens by using a digital hardness testing system with a load of 50 g. Three indents at each depth were measured to obtain the average hardness. The distance between two indents was kept at least 40 μm to ensure that the adjacent measurements were not affected by each other.

## 3. Results

### 3.1. Analysis of Microstructural Characteristics

#### 3.1.1. Misorientation Distribution

The kernel average misorientation (KAM) method was employed to characterize the extent of plastic deformation of the material. The KAM method is based on measuring the average misorientation of a point in a grain relative to its neighbors. The value of KAM reflects the variation of average misorientation in a grain. The higher value of KAM represents the larger amount of plastic deformation.

**Figure 4:**
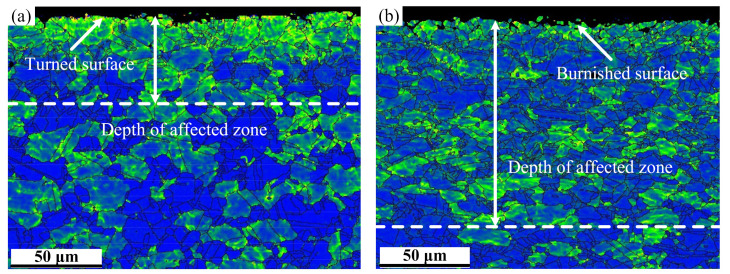
The evolution of the kernel average misorientation (KAM) map induced by (**a**) FT and (**b**) LPB processes.

Figure 4 depicts the evolution of the KAM map of specimens from the FT process to the LPB process. From the Figure 4a, high density of local misorientation occurs at the surface and subsurface for the FT process. The same phenomenon for the LPB process can be observed in Figure 4b. This indicates that the plastic deformation was generated at the surface and subsurface during the turning and burnishing processes. In contrast, low density of misorientation (blue area) appears in the bulk material. This can be attributed to the recovery and recrystallization that had taken place in the bulk material during forging, solution heat treatment and aging treatment, which result in the low density of misorientation.

**Figure 5:**
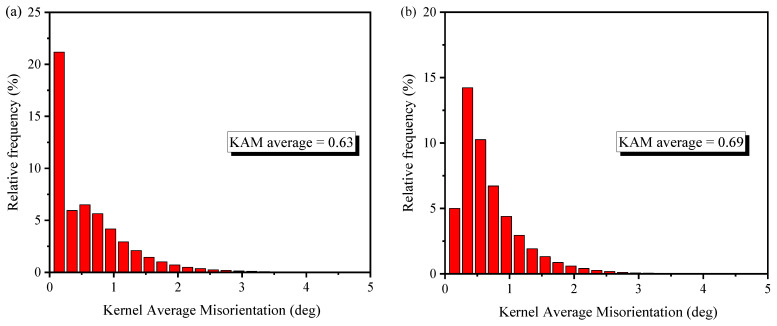
The average values of KAM after (**a**) FT and (**b**) LPB processes.

From the Figure 4, it is observed that the depth of the affected zone induced by the LPB process is deeper than that of the FT process, which indicates that the local misorientation increases after the LPB process. This increase between the FT and LPB processes can be further demonstrated from the average values of KAM, as shown in Figure 5. It is evident from Figure 5 that the LPB process shows a higher average value of KAM (0.69°) compared with the FT process (0.63°). The local misorientation distribution peak shifts towards higher misorientation angle from 0.15 ° to 0.35°. Therefore, the overall KAM distribution is shifted towards high angles after burnishing. The similar trend is also found in Lemarquis et al. [28], the experimental results showed that the local misorientation distribution peak shifts towards a higher misorientation angle from 1.02° to 1.26° after cold-burnishing AISI 316L. The higher values of KAM and DOA indicate that the LPB process induces a larger plastic deformation than the FT process.

#### 3.1.2. Misorientation Distribution

**Figure 6:**
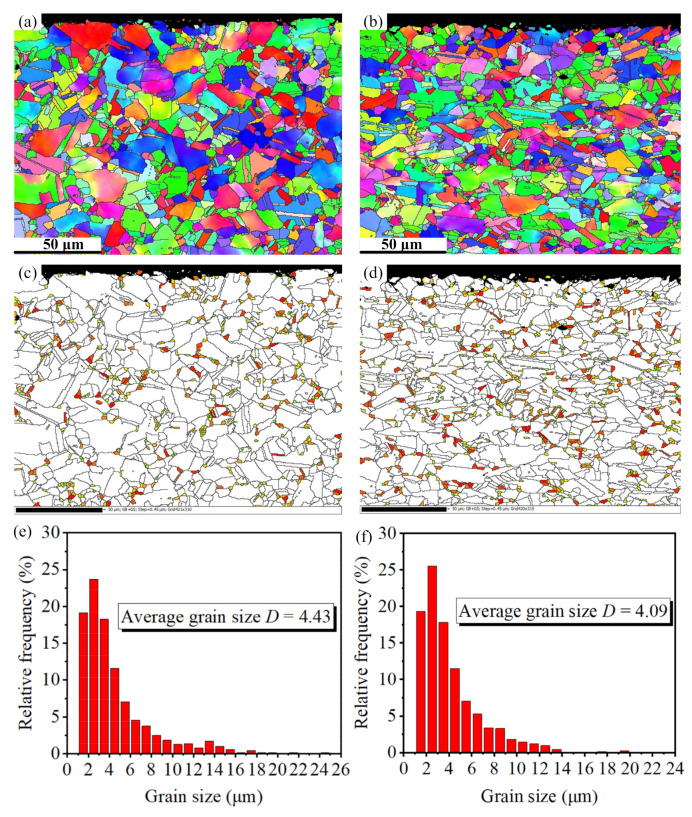
Grain size distribution after: (**a**,**c**,**e**) the FT process; (**b**,**d**,**f**) LPB processes.

During the LPB process, the grain size and boundary misorientation angle are two key parameters accompanied with the microstructural evolution, which affect the mechanical and fatigue properties of the material. Thus, it is necessary to investigate the grain size and boundary misorientation evolution after FT and LPB processes. The first parameter is grain size. The average grain size of FT and LPB specimens can be obtained through EBSD, as presented in Figure 6. In the Figure 6a,b, grain refinement can be clearly observed after the LPB process. There is an increase in the number of small grain (the diameter D < 3 μm) after the LPB process compared with the FT process, as presented in Figure 6c,d. The average grain diameter has a reduction from 4.43 to 4.09 μm after the LPB process compared with the FT process (Figure 6e,f)

One reason for the grain refinement is due to the occurrence of dynamic recrystallization (DRX). Dehmas et al. [29] demonstrated that the temperature at which DRX occurred was about 950 °C. The previous work [30] found that the temperature at the workpiece surface was about 750 °C when dry turning Inconel 718 under the feed rate of 0.1 mm/rev; LPB is classified as the cold-working process, hence the surface temperature is lower than 950 °C during burnishing. Thus, the temperature generated during FT and LPB are lower than the temperature for DRX occurrence. Consequently, the mechanism of DRX could not be contributed to the grain refinement during FT and LPB processes.

**Figure 7:**
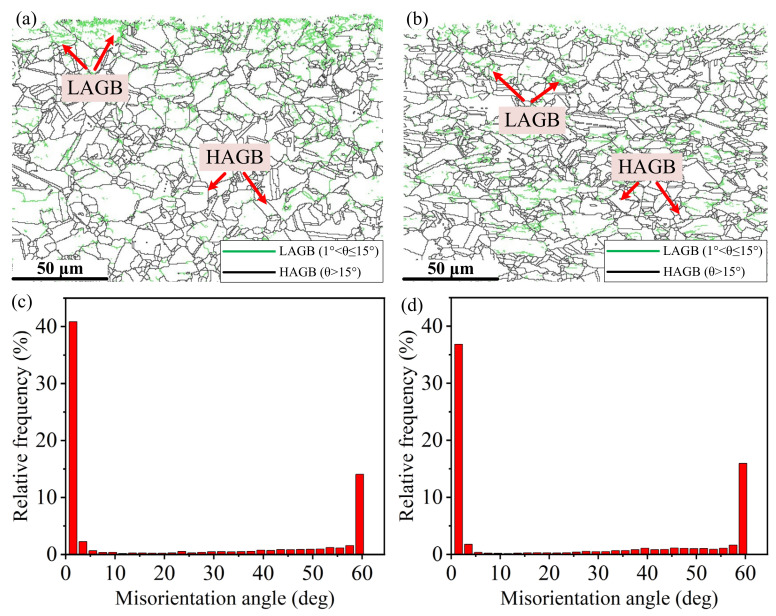
LAGBs and HAGBs distribution after: (**a**,**c**) the FT process; (**b**,**d**) the LPB process.

The second parameter is the boundary misorientation angle. Seita et al. [31] reported that grain boundaries could be contributed to the strengthening after SP treatment. The low angle grain boundaries (LAGBs) and high angle grain boundaries (HAGBs) distribution after FT and LPB processes are presented in Figure 7. The high density of LAGBs (1 < *θ* ≤ 15°) can be observed in the region near the top surface after the FT process. It is clear form the Figure 7a that LAGBs (1 < *θ* ≤ 15°) decrease significantly with the distance from the turned surface. After the LPB process, a number of LAGBs (1 < *θ* ≤15°) occur at the subsurface, which gives an indication of the increase in dislocation density at the subsurface.

**Figure 8:**
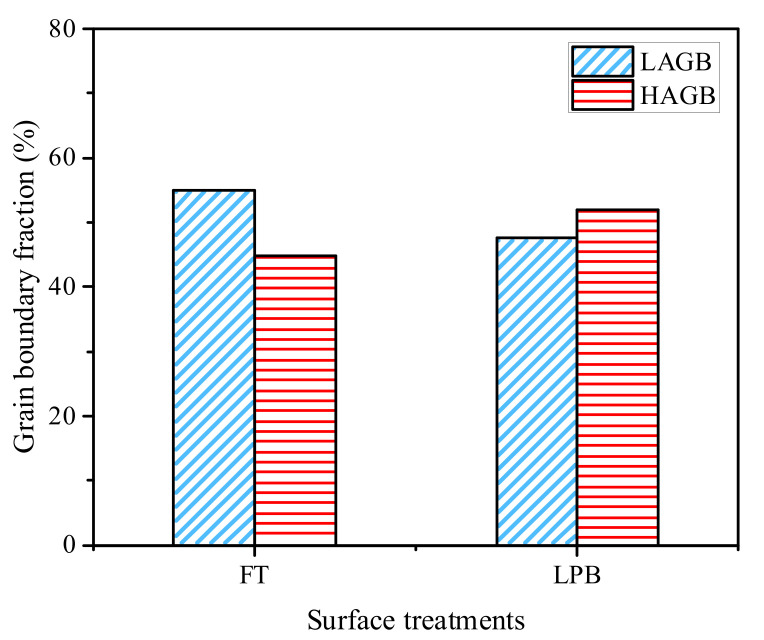
Grain boundaries fraction after (**a**) FT and (**b**) LPB processes.

However, it should be noted that there is a decrease in the fraction of LAGBs from 54.9 to 47.5% after the LPB process compared with the FT process, as shown in Figure 8. This phenomenon indicates that a certain number of LAGBs had been rotated during LPB, which evolved into HAGBs (*θ* > 15°). Chamanfar et al. [32] reported that the plastic deformation generated by SP would rotate the grain along the slip system, hence leading to the formation of HAGBs. Chen et al. [33] also proposed that the mutual rotation of grains and the grain boundaries slip induced the HAGBs. There are two main ways for the formation of HAGBs. One is induced by the microstructural evolution. The other is from the evolution of the texture during deformation. The increase in the boundary misorientation angle between adjacent grains is realized by more dislocation accumulation and annihilation at the grain boundaries, the mutual rotation of the grains, the grain boundaries slip and so on. However, the HAGBs produced by the microstructural evolution are generally within the range of 15~30° [34].

**Figure 9:**
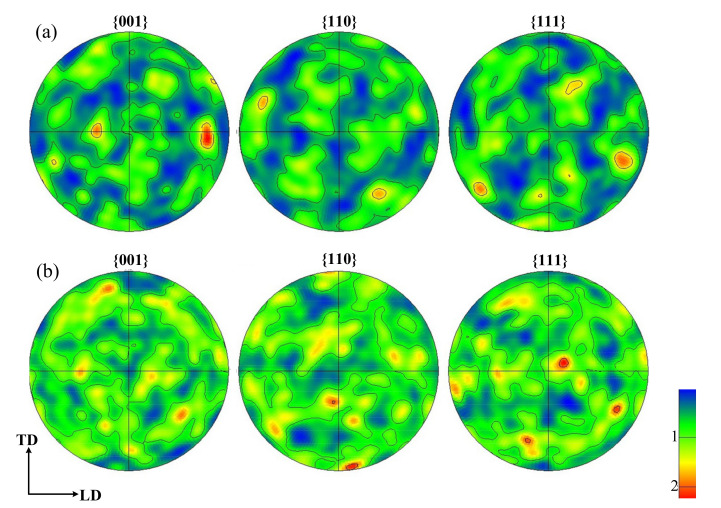
Corresponding pole figures after (**a**) FT and (**b**) LPB processes.

The evolution of texture also induces a large number of HAGBs. The pole figure reconstructed after FT and LPB processes is presented in Figure 9. After the FT process, the specimen shows a strong texture with {001} in the longitudinal direction. After the LPB process, the texture with {001} in the longitudinal direction tends to be weakened. In addition, the texture peak intensity is decreased after the LPB process, compared with the FT process, from 2.85 to 2.27, while keeping the same calculation parameters. Studies have shown that the cold-rolling process decreases the texture intensity of AISI 316L [28]. The decrease of texture intensity is responsible for the refined grains’ spin to different preferred orientations. These stable preferred orientations can vary greatly, resulting in HAGBs. Consequently, the grain refinement phenomenon can be explained as the increase of KAM within a grain and the formation of HAGBs due to the plastic deformation during the LPB process.

### 3.2. Micro-hardness

**Figure 10:**
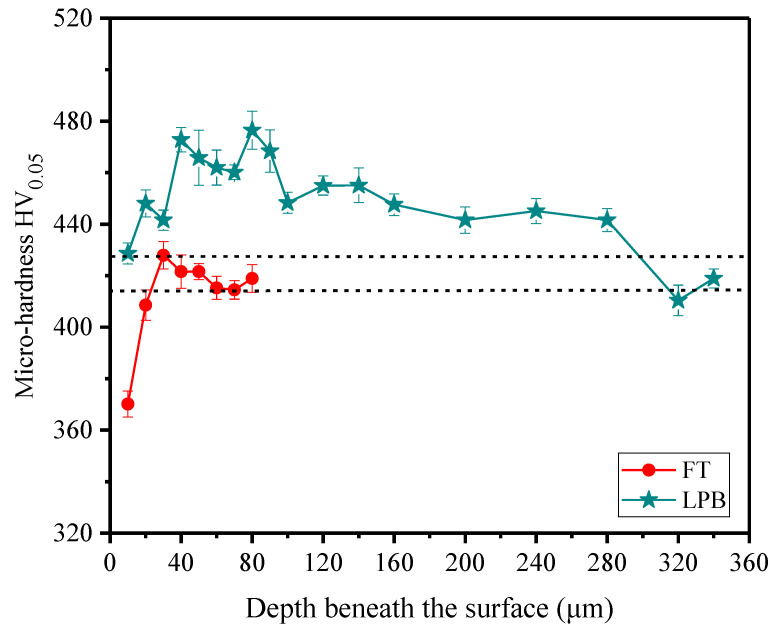
Micro-hardness distribution in depth beneath the surface.

The micro-hardness distributions in depth beneath the surface are shown in Figure 10. It can be observed that the micro-hardness at the surface is 370 HV_0.05_, which is lower than that of the bulk material after FT. This implies that a softening phenomenon occurs on the specimen surface. The phenomenon could be explained as the low thermal conductivity of Inconel 718, which causes a large amount of heat accumulated on the machined surface, resulting in lower hardness. The similar phenomenon was observed by Wu et al. [35], who found that a softening phenomenon was generated during finish turning Inconel 718. After LPB, the surface micro-hardness increases obviously from 370 to 429 HV_0.05_, and a deep hardening layer appears at the subsurface. The increase in micro-hardness can be related to the severe plastic deformation at surface induced by LPB.

### 3.3. Residual Stress

**Figure 11:**
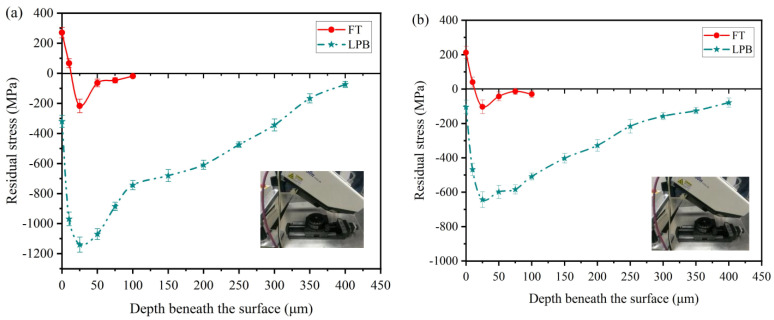
Residual stresses distribution in depth beneath the surface in (**a**) longitudinal and (**b**) transverse directions.

Residual stresses measured by using the XRD technique. The residual stresses distribution in depth beneath the surface after FT and LPB are shown in Figure 11. As seen from the figure, tensile surface residual stresses are generated in both longitudinal and transverse directions for FT, whereas LPB produces compressive surface residual stresses in these two directions. On the other hand, LPB induces the deeper depth of residual stress distribution, which means more plastic deformation is generated by LPB.

The peak compressive residual stress for the FT specimen is −217 MPa at the depth of 25 μm. After LPB, the peak compressive stress reaches −1136 Mpa, which is increased by a factor of 4.2 compared with FT. It is noteworthy that the peak compressive residual stress for LPB specimen is 91% of *σ*_0.2_. Klotz et al. [36] proposed that the peak compressive residual stress occurred at the depth of 20 μm, and the peak value was 88% of *σ*_0.2_ after shot-peened Inconel 718. It is indicated that LPB induces the higher peak compressive residual stress compared with SP. As exhibited in Figure 11, the residual stress distribution in the longitudinal direction is more severe than in the transverse direction. The anisotropy in residual stress distribution can be attributed to the difference in the amount of plastic deformation that generated along different directions.

### 3.4. Plastic Strain

**Figure 12:**
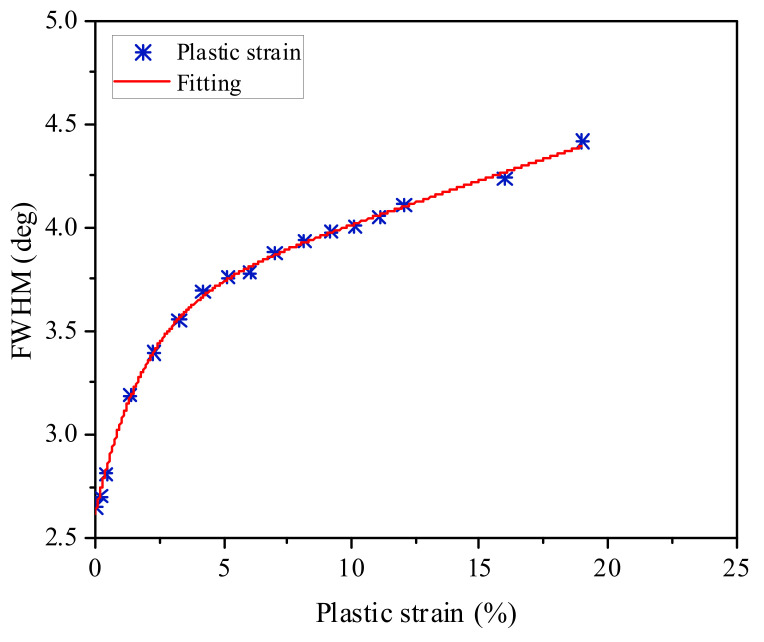
The relationship between full width at half the maximum (*FWHM*) and plastic strain.

Prevéy et al. [37] have demonstrated that the diffraction peak width was associated with local plastic strain of treated material; Klotz et al. [36] have also verified this relationship by conducting the similar experiments. The relationship between the full width at half the maximum (FWHM) and plastic strain can be expressed as:

(1) $$FWHM=A\left[1-e^{B\varepsilon_{p}}\right]+C\varepsilon_{P}+K$$

where *A*, *B*, *C* and *K* are the parameters that determined by non-linear least square regression; *ε_p_* is the plastic strain. For Inconel 718 superalloy, the calibrated *FWHM* versus plastic strain curve was obtained, as presented in Figure 12. According to the fitting curve, the parameters *A*, *B*, *C* and *K* are 0.962, 0.481, 0.041 and 2.65, respectively.

**Figure 13:**
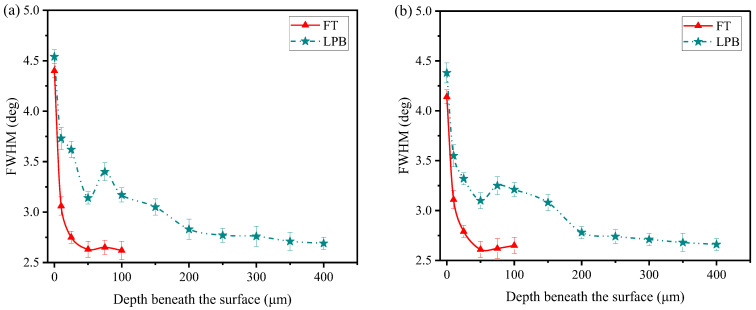
FWHM distribution in depth beneath the surface in the (**a**) longitudinal direction and (**b**) transverse direction.

*FWHM* is measured in longitudinal and transverse directions by using XRD diffraction, and is summarized in Figure 13. It can be observed that *FWHM* is a function of depth after FT and LPB. The maximum value of *FWHM* occurs at the surface and decreases gradually with the increase of depth. A similar *FWH*M distribution was observed by Kattoura et al. [38], who reported that the value of *FWHM* was higher at surface (4.5°) and reduced gradually to a constant through the depth.

**Figure 14:**
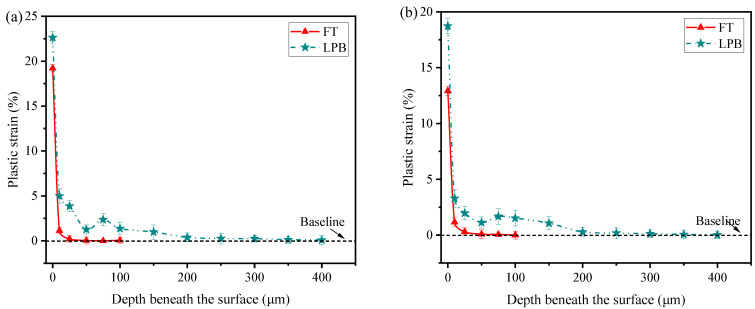
Plastic strain distribution in depth beneath the surface in the (**a**) longitudinal direction and (**b**) transverse direction.

The plastic strain is calculated according to the Equation (1). Figure 14 presents the plastic strain distribution in longitudinal and transverse directions. As depicted in Figure 14a, FT induces 19.22% plastic strain at surface and the depth of plastic strain is 50 μm; while LPB produces higher plastic strain (22.63%) at surface and a deeper depth of plastic strain (400 μm). The depth of plastic strain distribution seems to be similar to the depth of residual stress distribution. From Figure 14b, it can be seen that the plastic strain distribution in the transverse direction is similar to that in the longitudinal direction. Nevertheless, it should be noted that both FT and LPB induce higher plastic strain in the longitudinal direction than in the transverse direction. This indicates that a larger amount of plastic deformation was generated in the longitudinal direction during FT and LPB, which is supported by the anisotropy in residual stress distribution, as shown in Figure 11.

### 3.5. Tensile Properties

**Figure 15:**
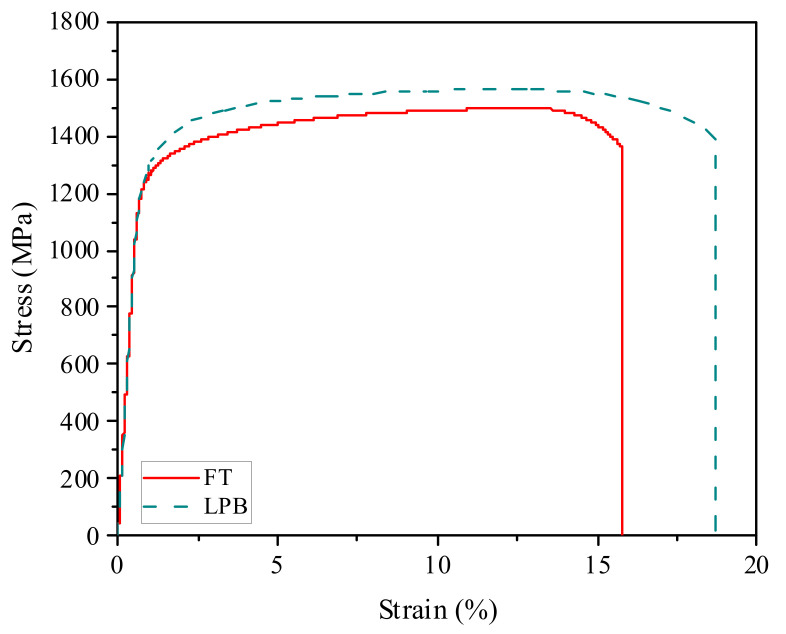
Stress–strain curves of Inconel 718 for FT (red) and LPB (green) processes.

Uniaxial tension tests were performed to evaluate the tensile properties of Inconel 718. Figure 15 presents the stress–strain curves of Inconel 718 for FT and LPB at room temperature. It is clearly seen that the yield strength is improved from 1210 to 1242 MPa, and the ultimate tensile strength is improved from 1495 to 1563 MPa after the LPB process. The similar increase was also observed in the laser shock peening (LSP) Inconel 718 by Kattoura et al. [39], but they found that the failure strain was reduced by 12% after LSP treatment compared with the un-peened specimen. They concluded that the decrease failure strain was attributed to the increase of hardening effect. After the LPB process, the failure strain is increased by 18.5%. This seems to indicate that LPB induces a lesser hardening effect compared with LSP, resulting in less relaxation of compressive residual stresses.

Comparison of the current study with the ultrasonic nanocrystal surface-modified [38] and laser shock peened 718 alloy [39] are summarized in Table 3. It is clear that all the tensile properties of the LPB specimen are higher than that of the FT specimen. This phenomenon can be associated with the evolution of microstructure induced by LPB treatment. The yield strength ratio *σ*_0.2_/*σ*_b_ represents the plasticity of the material. It is noteworthy that the yield strength ratio *σ*_0.2_/*σ*_b_ of the LPB specimen is lower than the LSP specimen. This indicates that the LPB specimen has better plasticity than that of the LSP specimen. Compared with the UNSM process, the LPB process induces a higher value of yield strength. Nevertheless, the yield strength ratio of the LPB specimen is higher than that of the UNSM specimen. This means that the LPB process produces a larger amount of plastic deformation, resulting in a higher degree of microstructural evolution at the surface layer.

### 3.6. Strengthening Mechanism Induced by the LPB Process

According to the dislocation structure model of low-angle grain boundaries (*LAGBs*), the dislocation density (*ρ_LAGB_*) in the *LAGBs* can be calculated as follows [40]:

(2) $$\rho_{LAGB}=\frac{1.5\theta S_{v}^{LAGB}}{b}$$

where *b* is the Burgers vector, *θ* is the boundary misorientation angle, and $S_{v}^{LAB}$ is the *LAGBs* area per unit volume.

Based on the Taylor model, the relationship between the dislocation density and material strengthening can be expressed as:

(3) $$\sigma_{dis}=\alpha MGb\sqrt{\rho }$$

where *α* is a constant (for the metal of the FCC crystal structure, α = 0.24), *M* is the Taylor factor, *G* is the shear modulus with the relationship G=E/2(1+μ), and *ρ* is dislocation density.

Therefore, the contribution of *LAGB*s and dislocation to material strengthening can be written as:

(4) $$\sigma_{f}=\alpha MGb\sqrt{\rho_{LAGB}+\rho_{0}}$$

where *ρ*_0_ is the density between the boundaries. As the value of *ρ*_0_ is relatively small, its contribution to strengthening may be neglected. Consequently, the contribution of LAGBs can be rewritten as:

(5) $$\sigma_{LAGB}=\alpha MG\sqrt{1.5b\theta S_{v}^{LAGB}}$$

According to the Hall–Petch equation, the contribution of high-angle grain boundaries (*HAGBs*) to material strength can be expressed as follows [41]:

(6) $$\sigma_{HAGB}=k_{HP}{\left(D_{grain}\right)}^{-\frac{1}{2}}$$

where *D_grain_* is the average grain size and *k_HP_* is a material dependent constant.

After burnishing processes, the deformation structure contains a large number of *LAGBs* and *HAGBs*, thus the strength of Inconel 718 after the LPB process can be rewritten as the sum of contributions of *LAGBs* and *HAGBs*, as follows:

(7) $$\sigma =\sigma_{0}+\sigma_{LAGB}+\sigma_{HAGB}=\sigma_{0}+\alpha MG\sqrt{1.5b\theta S_{v}^{LAGB}}+k_{HP}{\left(D_{grain}\right)}^{-\frac{1}{2}}$$

By introducing the total surface area of all the boundaries per unit volume *S_v_* and the fraction of high angle grain boundaries, *f_HAGB_*, *ρ_LAGB_* and *D_grain_* can be written as follows [41]:

(8) $$\rho_{LAGB}=\frac{1.5\theta S_{v}\left(1-f_{HAGB}\right)}{b}$$

(9) $$D_{grain}=\frac{D_{av}}{f_{HAGB}}$$

where *D_av_* is the average spacing between all boundaries and with the relationship *D_av_* = 2/*S_v_*. Hence, the Equation (8) can be rewritten as:

(10) $$\rho_{LAGB}=\frac{3\theta \left(1-f_{HAGB}\right)}{bD_{av}}$$

Therefore, the Equation (7) can be rewritten by the following equation:

(11) $$\sigma =\sigma_{0}+\left[\alpha MG\sqrt{3b\theta \left(1-f_{HAGB}\right)}+k_{HP}\sqrt{f_{HAGB}}\right]{\left(D_{av}\right)}^{-\frac{1}{2}}$$

According to the Equations (7) and (11), it can be found that the enhanced contribution of the deformable structure in Inconel 718 mainly comes from the high angle grain boundaries, low angle grain boundaries, and the average spacing between all boundaries. Thus, the value of strength tends to be higher as the fraction of LAGBs and the average spacing decrease after the LPB process.

## 4. Conclusions

In the present work, low plasticity burnishing was employed to modify the surface and subsurface characteristics of Inconel 718. The evolution of microstructure and mechanical properties was investigated. The main conclusions can be drawn as follows.

(1) After the LPB process, the average grain diameter decreased from 4.43 to 4.09 μm, and the fraction of LAGBs decreased from 54.9 to 47.5%. The grain refinement phenomenon could be attributed to the increase of KAM and the formation of HAGBs.
(2) The peak compressive stress was increased by a factor of 4.2 after the LPB process. The LPB process produced higher plastic strain (22.63%) at the surface, which indicated that a larger amount of plastic deformation was generated during the LPB process.
(3) The LPB process induced higher values of the yield strength and the ultimate tensile strength. The enhanced yield strength depended on the average spacing between all boundaries and the HAGBs.

Further research is recommended to be performed to quantify the influence of microstructure on fatigue life for various service requirements and to relate the characterization of microstructure to different processing conditions. It is important to control the evolution of microstructure for improving the mechanical properties and fatigue life.

## Figures and Tables

**Table 1:** Chemical composition of Inconel 718 (% wt).

Ni	Cr	Mn	Nb	Mo	Ti	Al	Cu	Si
53.51	18.05	0.062	5.43	2.98	1.02	0.5	0.035	0.074
**C**	**Co**	**P**	**Ta**	**B**	**Ca**	**N**	**Mg**	**Fe**
0.025	0.31	0.01	0.0085	0.0042	0.0032	0.0079	0.0014	Bal

**Table 2:** X-ray diffraction parameters used for residual stress measurements.

Wavelength	Tube Target	Diffraction Plane	Bragg Angle (2θ)
Kβ (λ = 2.085 Å)	Cr	{311}	150.89°
**Tube voltage**	**Tube current**	**X-ray slit**	**Exposure time**
30 kV	1 mA	2 mm	90 s

**Table 3:** Tensile properties of different treatments.

Treatment	Yield Strength	Ultimate Tensile Strength	Elongation (%)	Yield Strength Ratio *σ*_0.2_/*σ*_b_
	*σ*_0.2_ (MPa)	*σ*_b_ (MPa)		
FT	1210	1495	15.7	0.81
LPB	1242	1563	18.7	0.79
UNSM [38]	1217	1677	21.1	0.73
LSP [39]	1288	1602	22.1	0.8

## Data Availability

Not applicable.
